# Privacy and Well-Being in Aged Care Facilities with a Crowded Living Environment: Case Study of Hong Kong Care and Attention Homes

**DOI:** 10.3390/ijerph15102157

**Published:** 2018-10-01

**Authors:** Yiqi Tao, Stephen Siu Yu Lau, Zhonghua Gou, Jiayan Fu, Boya Jiang, Xiaowei Chen

**Affiliations:** 1 Department of Architecture, National University of Singapore, Singapore 117566, Singapore; taoyiqi@gmail.com (Y.T.); laustephensyy@gmail.com (S.S.Y.L.); 2 School of Engineering and Built Environment, Griffith University, Gold Coast, QLD 4215, Australia; 3 Department of Architecture, College of Civil Engineering and Architecture, Zhejiang University, Hangzhou 310000, China; fujiayan513@gmail.com; 4 School of Architecture, Nanjing Tech University, Nanjing 211816, China; jiangboya@njtech.edu.cn; 5 School of Public Affairs, Zhejiang University, Hangzhou 310000, China; florachenxiaowei@zju.edu.cn

**Keywords:** privacy, well-being, elderly, aged care facilities, compact living

## Abstract

This study aims to understand the relationship between bedroom privacy and well-being of the elderly in aged care facilities with a compact living situation. A majority of studies on this topic were carried out in a low-density population context. The crowded living situation might compromise the well-being of residents. This study proposed five architectural parameters to measure bedroom privacy in aged care facilities: total open surface per unit, openness/solid ratio per bed, height of partition wall, number of people per unit, and personal control over bedroom privacy. SF-12 v.2 Health Survey was used to collect information on physical and mental health status. The study surveyed nine Care & Attention homes and their 213 residents in Hong Kong. The total open surface per unit and the openness/solid ratio per bed were positively associated with the physical health of residents. The height of partition walls was associated negatively with their physical and mental health conditions, and the number of people per unit was negatively associated with their physical health. More than half of respondents preferred a single unit with high partition walls; however, 40% of respondents preferred low partition walls. The provision of privacy for the elderly should be balanced with their needs for social interactions; total open surface per unit, openness/solid ratio per bed and height of partition wall should be taken into consideration. The study provides evidence and design guidelines for improving privacy in aged care facilities with a compact living environment.

## 1. Introduction

### 1.1. Privacy

Privacy is an important human need, which can be viewed as a process of boundary control available to be exercised by the individual to manage both personal and social interactions [1]. Westin [2] defined privacy as “the claim of individuals, groups, or institutions to determine for themselves: when, how, and to what extent the information about them is communicated to others”. Other similar definitions have referred to the adoption of visual and auditory obstacles to avoid interaction and intrusion and the ability to control unwanted interactions [3]. As per the context for privacy, Altman distinguished a number of relationship types: person-to-person, person-to-group, group-to-person, and group-to-group [4]. The maintenance of individual privacy could benefit the individual in personal autonomy (through the avoidance of manipulation or domination by others), emotional release (meeting physical and psychological health demands), self-evaluation (integrating one’s experience into a meaningful pattern and exerting one’s individuality on events), and limited and protected communication (providing an opportunity for sharing intimacies whilst also setting necessary boundaries [2].

In long-term care settings such as nursing homes, personal space and territory have been identified as two key characteristics of the physical environments for promoting the well-being of elderly residents [5,6]. As observed by Altman [4], personal space is a mechanism used to regulate interpersonal interaction and to achieve a desired level of privacy. In nursing homes, the residents’ territory means their bedrooms. The bedroom represents a private area away from the demands of others and allows the individual to pursue his or her independence [7,8]. It has been emphasized that, if the impact of physical environment on elderly people is to be understood, personal space and territoriality must be taken into consideration [9]. Health professionals in a variety of institutions, but particularly in nursing homes, must respect the concept of territoriality, which figures prominently amongst the physical and psychological needs of their clients [10]. Kane et al. [11] believed that residents’ right to privacy is one of the regulatory quality of life expectations in nursing homes. Applegate and Morse [12] argued that an individual’s expectation and experience of privacy in nursing homes was influenced by both personal and environmental factors. Leino-Kilpi et al. [13] also highlighted that the most common privacy problems in nursing environments have to do with noise, limited space, and restrictions.

### 1.2. Crowding

One of the parameters most critically influencing individual privacy is crowding, which occurs when privacy fails to function successfully. In a crowded environment, more interaction takes place than is desired, causing an imbalance between desired and achieved levels of privacy [4,14]. According to Stokols [15], the perception of crowding might result in stress. In psychological terms, the stress may take the form of cognitive inconsistency, reflecting the discrepancy between the space desired and the space actually available; physiological symptoms may include hormonal secretions and a rise in blood pressure. Other researchers have reported similar findings. Evans [16], for example, observed that residential crowding (measured by the number of people per room) would elevate psychological distress and that people who had private bedrooms were better adjusted and more socially engaged than those who had not. One empirical survey conducted in the UK indicated the internal crowding (dwelling space per person) influenced psychological health in women [17]. Another study discovered that residential density could trigger psychological distress two months after admission [18]. Schopp et al. [19] pointed out that an invasion of a resident’s privacy attributed to instances where too many roommates have to share sleeping space or where the building design and layout fails to provide space for residents to spend time alone, especially in double or multi-bed rooms.

Respect for privacy is considered as a routine part of nursing care and a moral obligation, and indeed, it has been claimed as a legal requirement of institutional health care. However, it has been reported that when elderly people were admitted to institutional facilities, the consequence of the loss of privacy was significant [20]; this was compounded by a loss of possessions [10]. Research into privacy in hospitals showed that the elderly were more likely to lose privacy than young patients [21]; while a similar study also revealed that institutional environments often required seniors to compromise their perceptions of privacy, territory, and boundaries [7].

In sum, understanding the elderly’s privacy in a crowded living environment is an important research issue for designing healthy built environments. This study, therefore, has been conducted to investigate the elderly residents’ bedroom privacy in aged care facilities with crowded living environments, to find whether a relationship exists between bedroom privacy and the well-being of elderly residents, and finally to identify a set of design parameters that can predict privacy and well-being for residents in aged care facilities.

## 2. Methods

### 2.1. Care and Attention Homes

**Figure 1:**
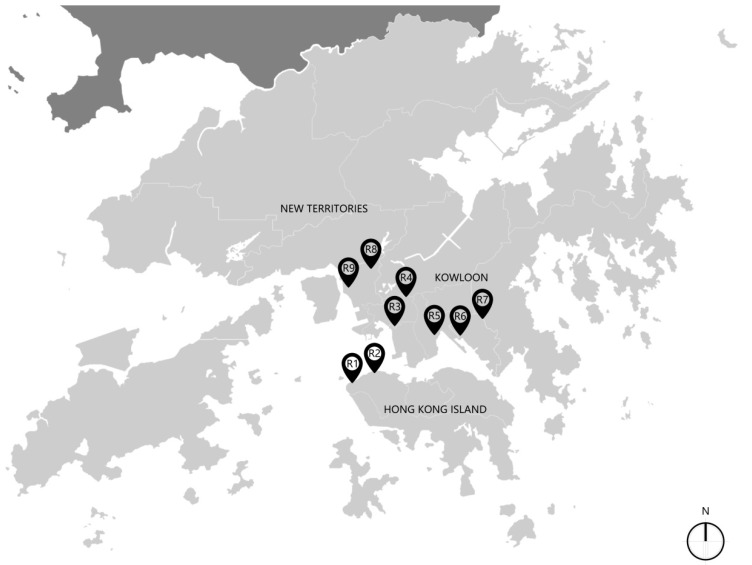
Locations of the nine Care and Attention (C&A) homes in Hong Kong (drawn by the authors).

**Figure 2:**
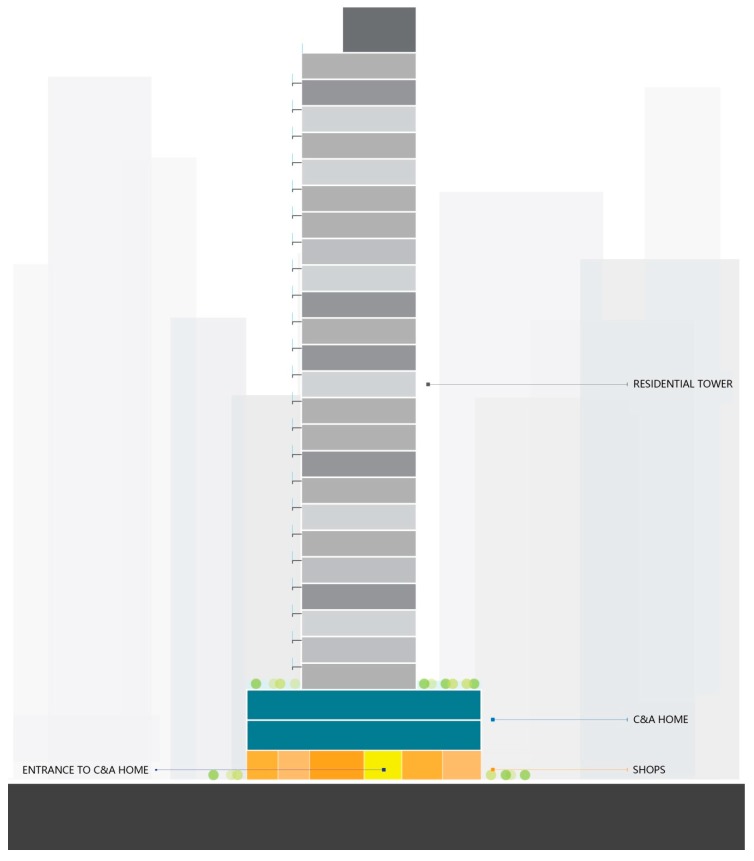
C&A homes in tall buildings (drawn by the authors).

This study was conducted in Hong Kong in January 2016 and involved nine Care and Attention (C&A) homes (R1–R9) with compact living conditions. C&A homes are the main aged care facilities in Hong Kong for the aged adults with poor physical ability or mild mental disability. The service provides residential care, meals, and a limited degree of nursing care. The C&A homes adopt a compact living policy due to land and resources limitation [22]. The mean bedroom area of these investigated C&A homes is 4 m^2^. All C&A homes were located at podium levels (lower levels in the residential tower, usually for commercial purpose such as supermarkets and canteens, the area of which is usually larger than the residential unit) of residential towers in a high-rise and high-density residential district. (Figure 1 and Figure 2).

**Figure 3:**
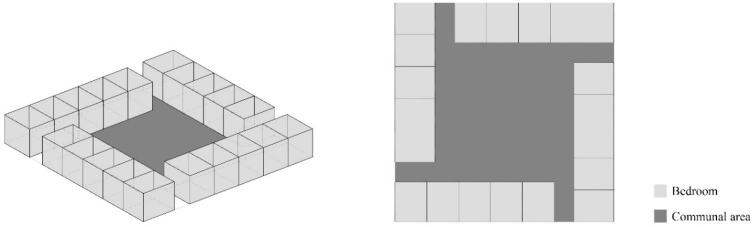
The arrangement of bedroom and communal areas (drawn by the authors).

**Figure 4:**
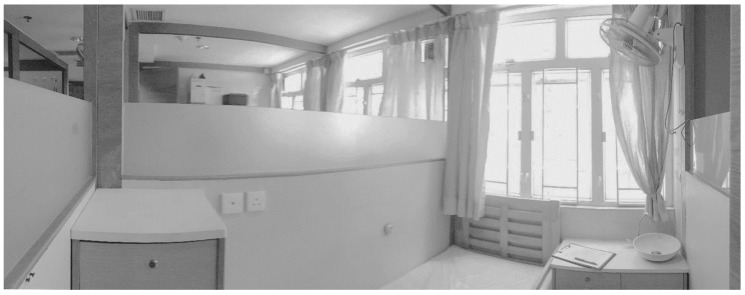
Typical chamber of C&A homes in Hong Kong (photo by the authors).

**Figure 5:**
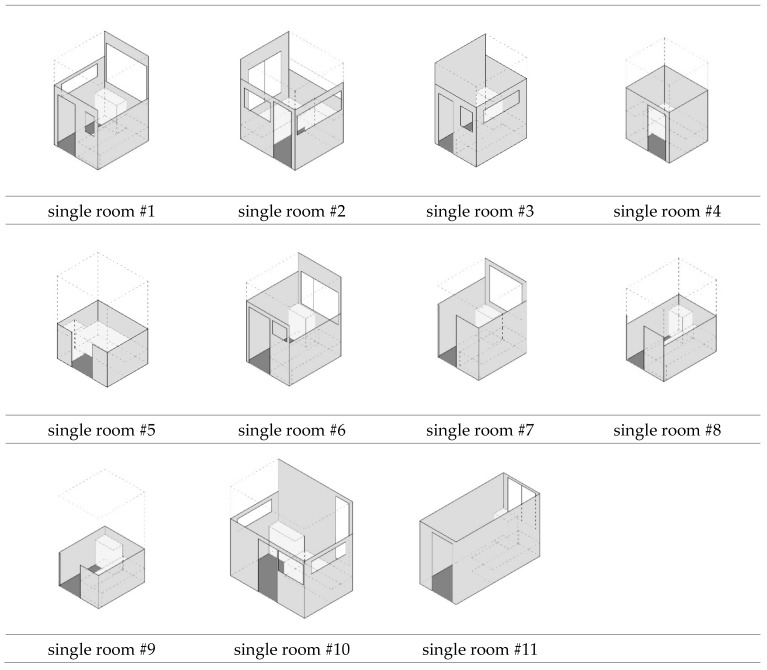

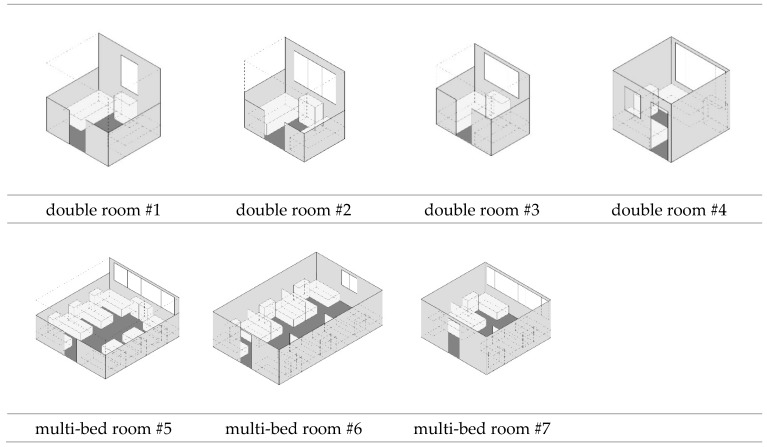
Axonometric view of the bedrooms (drawn by the authors).

On the typical residential floor, the communal area (living room) is located in the center, with the personal chambers (bedrooms) along the outer walls of the building (Figure 3). Their unique chamber-type bedroom design emerged in response to the reality of a high-density living environment, with small chambers carved out of a large space and separated not by solid walls but by partition walls (Figure 4). These partition walls are generally made of wood panels and provided a measure of privacy; all of these walls are not erected to the ceilings; the height and design of partition walls vary from one to another C&A homes (Figure 5). Each chamber was equipped with a single bed (a few with two beds) and a bedside table for daily storage.

### 2.2. Measurement of Privacy Related Architectural Parameters

In architectural terms, privacy is a measurable quality that could be quantified in terms of the size, height, and character of a wall [7]. Franz [23] concluded that the size and rate of the enclosure are probably two primary aspects that affect architecture space. A more recent study by Indraprastha and Shinozaki [24] has noted visual openness, privacy, and physical accessibility as parameters that might influence the quality of an enclosed space. It also revealed that the level of privacy experienced in a space depended mainly on two parameters: openness (the ratio of the open area to its adjacent wall area) and circulation gate (the average distance from the center of the enclosed space to the door). This study involved both physical measurements of privacy-related design parameters and a questionnaire survey of the residents.

**Figure 6:**
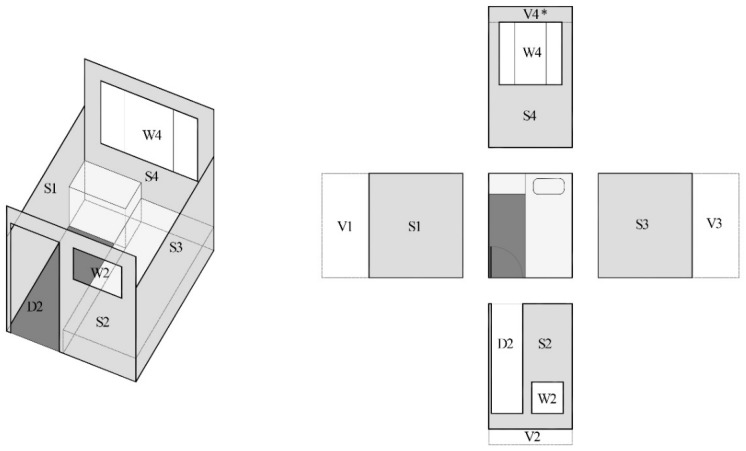
Typical bedroom unit for the calculation (drawn by the authors).

In the present research, five privacy related design parameters are proposed for the measurement: total open surface per unit, openness/solid ratio per bed, height of partition wall, number of people per unit, and personal control over bedroom privacy. These five parameters are selected based on the literature. Openness (open surface) is a key parameter for the privacy control. Most of the parameters are designed to exam the openness in a different way which could easily reflect the existing bedroom conditions in C&A homes. The first four parameters, in different respects, measure the degree of exposure of a resident in his or her unit. The measurement is based on an on-site survey using a laser distance meter, LDM-40, with an accuracy of 1.5 mm (0.06 in), and the calculation is based on drawing analyses of the nine C&A homes and their bedroom units. Most C&A homes adopted a single bedroom design, with a few adopting double or multi-bed rooms. The open surface per unit mainly includes open areas such as windows, doors, and other void surfaces. In a typical single bedroom, the partition wall is lower than the ceiling; and the open area above the partition wall and below the ceiling is counted as a void surface in the calculation. Figure 6 shows a typical unit as a conceptual model for the calculation. The total open surface, inclusive of all void surface in the four elevations of a unit, is calculated based on V_total_ = V1 + V2 + V3 + V4* where V4* is only applied when the chamber is not sited against the external wall (when the chamber is against wall, there is no open surface above the partition wall; when the chamber is not against wall, these bedrooms do not have windows to the outside). The window (W) and door areas (D) are too regarded as open surface. Thus, the total open surface equals V_total_ + W_total_ + D_total_. The solid surface includes the total solid area on the four elevations, such as walls and partitions; so S_total_ = S1 + S2 + S3 + S4. The openness/solid ratio equals to (V_total_ + W_total_ + D_total_)/S_total_. The calculations for the double and multi-bed rooms adopt the same equation, with space equally being divided according to the number of beds (The term per unit stands for the unit which has only one bed and the term per bed stands for the unit which has more than one bed). C&A homes may have different styles of chambers, with different partition wall heights. In the following context, the height of the partition walls is calculated as a weighted mean value.

The fifth privacy parameter measured in this study, namely personal control, refers to how the residents maintain or control their privacy in the C&A homes. Personal control has been identified as a psychological concept crucial to privacy, and primarily concerns one’s behavior selection [25]. In this study, the personal privacy adjustment level was classified as (2) strong, (1) weak, or (0) non-controllable. Since research into hospital privacy has revealed that rooms with curtain/blinds walls provide significantly less auditory, visual, and overall privacy than rooms with solid walls [26], hard and non-transparent barriers like doors, which the users could operate to carve out private space from the external environment, were classified as providing a strong privacy adjustment level. Soft and transparent barriers like curtains or blinds permitted user adjustment and therefore could provide a relatively private space from the external environment; however, since these still admitted shadows, signs of movement, and the sounds of conversation from outside, these were classified as a weak privacy adjustment level. Finally, a personal chamber without doors, curtains, blinds, or other decorations capable of being used to adjust the privacy level was defined as a non-controllable environment and scored zero points for privacy control.

### 2.3. Questionnaire Survey of Health Status

The SF-12 v.2 Health Survey as a widely used evaluation tool for health status was included to collect information on overall physical and mental health outcomes. The SF-12 v.2 Health Survey contains eight domains, which include physical functioning, role-physical, bodily pain, general health, vitality, social functioning, role-emotional, and mental health. Each respondent answered 12 core questions in the survey. Based on individual responses, one physical health score (PSC) and one mental health score (MSC) is generated for each respondent. The range for each of these scores is from 0 to 100, and the mean score is 50. In the survey of 2410 adults in Hong Kong, Lam [27] used the SF-12 v.2 Health Survey and compared it with the full version SF-36; it was found that the SF-12 Health Survey v2 could explain 82–84% of the total variances of the full version and, therefore, it is valid, reliable, and applicable in Chinese contexts.

The sampled participants were identified as clear-headed individuals who would be able to answer the questionnaires independently. Participants indicated their preference for room types (single room, double room, and multi-bed room) and partition walls (low, high, and no preference). Other basic information collected from residents included age and gender. Each questionnaire interview lasted around 15 minutes by architecture students who speak Cantonese. Before the survey, they all trained on how to help respondents to correctly respond to the questionnaire. At the end of the survey, the respondents were given small gifts such as handkerchiefs as a token of thanks for their participation in the study. The questionnaire was approved by the Human Research Ethics Committee for Non-Clinical Faculties, HKU (Reference No.: EA1508015). After the measurement and questionnaire, regression analysis was conducted by IBM SPSS 20.0 (IBM Corp., Armonk, NY, USA), for analyzing the correlation between the privacy parameters and health-related quality of life.

## 3. Results

As shown in Table 1, a total of 213 occupants from nine C&A Homes participated in the survey, including 75 males and 138 females. The study group comprised approximately twice as many women as men. More than 40% of the respondents were over 85 years old; 28% were aged 75–84, and 30% were aged 65–74. All the participants were active aged adults who were capable of walking in the facility; bedridden residents were excluded from this study. On average, the SF-12 mean score for physical health was 41.8, and for mental health, it was 50.8.

The mean values of the five privacy related design parameters measured in the nine C&A homes are listed in Table 2. Linear and quadratic regression were conducted to model the relationship between physical health and mental health as dependent variables and privacy-related design parameters as explanatory variables. The result (Table 3) indicates that parameters such as the total open surface per unit and openness/solid ratio per bed could affect the physical health of occupants, with the optimal value of 11.5 m^2^ and 0.57, respectively. The linear regression indicated the height of partition walls associated negatively with both physical and mental health conditions; while the quadratic regression revealed that the optimal value for the height of the partition wall was 1.85 m. The number of people per unit parameter had a negative influence on the physical health of the occupants. Based on the quadratic regression analysis, there should be no more than seven people per unit.

**Figure 7:**
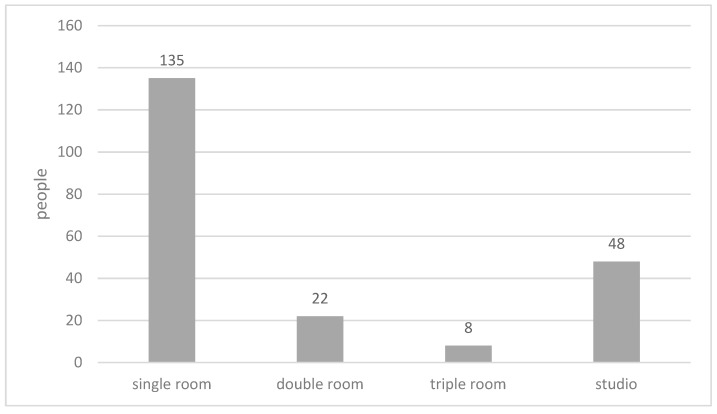
Preferred room types.

**Figure 8:**
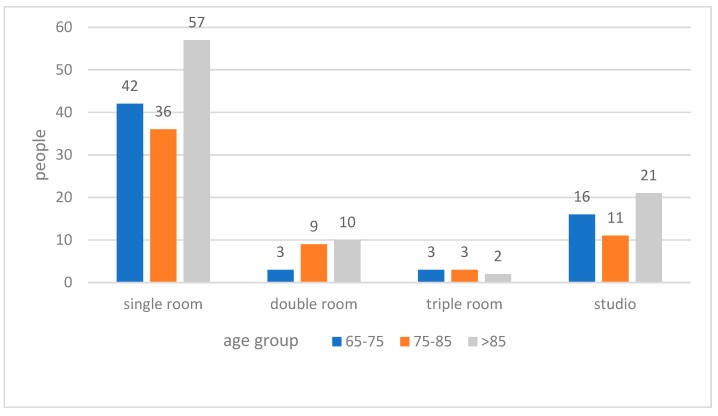
Preferred room types with age group.

When being asked about their preferred types of bedroom (Figure 7), 63% of the respondents of the total sample indicated that a single room would be their first choice. About 10% respondents of the total sample preferred living in a double room; while 27% respondents of the total sample indicated that they would like to live in a multi-bed studio in a large space. No correlation was found between the age group and preferred room types (Figure 8).

**Figure 9:**
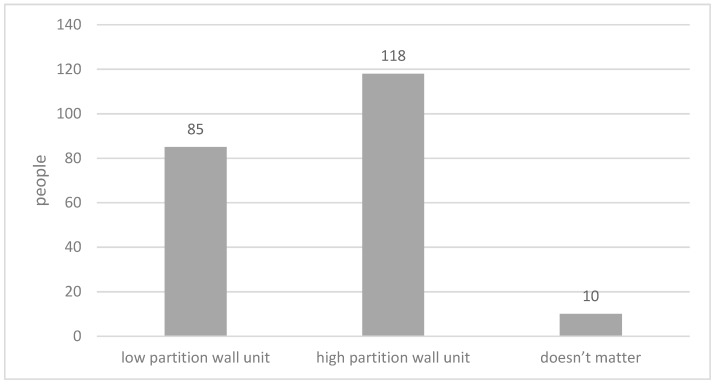
Preferred partition walls.

**Figure 10:**
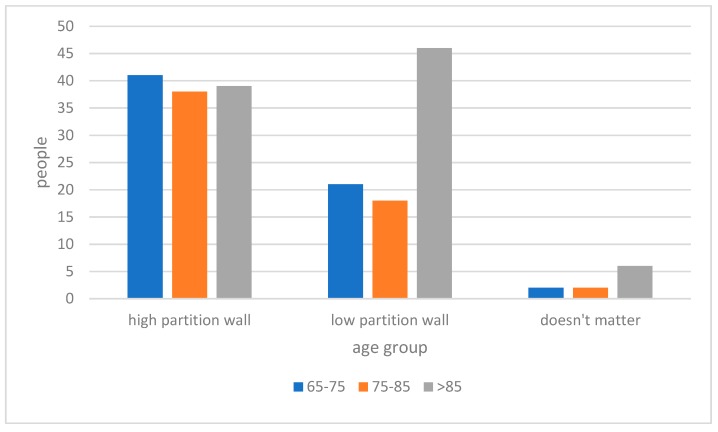
Preferred partition walls with age group.

As per the height of partition walls (Figure 9), the majority of respondents expressed a preference for a high partition wall unit, and 40% for a low partition wall unit; only 5% of respondents indicated that they had no preference in this matter. However, a significant portion of respondents who are above 85-year old preferred low partitions (Figure 10). The correlation analysis between age group and preferred height of the partition wall indicated the preferred height of the partition wall decreases as the age of respondents grows (Pearson Correlation: −0.183; *p* < 0.01).

## 4. Discussion

The crowded living situation in aged care facilities might compromise the well-being of residents. The results support the argument that in aged care facilities, a crowded living environment would influence the well-being of the residents. This, in turn, raises the question of how adequate privacy might be provided for residents living in a crowded environment like Hong Kong C&A homes. This study proposes five architectural parameters that were shown to be influential predictors for bedroom privacy in relation to the elderly well-being.

### 4.1. Total Open Surface per Unit

In this study, the linear regression revealed that the total open surface per unit (absolute value) had a positive influence on the state of an occupant’s physical health. Previous research had revealed that adequate window area might be especially important to individuals who spent a comparatively long time in the same room [28], and a greater volume of visual and acoustic exchange with the outside environment might have a significant positive effect on their health significantly. Lu et al. [29] too, revealed that the subtle difference in patient room visibility might have important effects on clinical outcomes, which should be emphasized by the architects. In C&A homes, elderly residents might be accommodated in bedrooms that afford an outside view and allow them to talk to the person in the next chamber through an open area above the partition walls. The proper total window surface per unit is one which balances the resident’s different needs. Adequate privacy must be provided without compromising their social interactions. Based on the finding of quadratic regression, the optimal open surface per unit was around 15 m^2^, which is a useful reference to the future design of C&A bedrooms.

### 4.2. Openness/Solid Ratio per Bed

In general, a large open surface would interfere with a resident’s sense of privacy; while a small one would have a deleterious effect on the resident’s health status. In this study, the quadratic regression indicated the ideal value of window/wall ratio of 0.57. Future designs should take the openness/solid ratio into consideration and balance the potential conflict of privacy and connections in a positive way.

### 4.3. Height of Partition Wall

Although the living space per person was quite small, most of the respondents in this study still reported a preference for a relatively private personal space (single chamber) with a high partition wall, to enjoy a degree of privacy. However, 40% of respondents, in contrast, indicated that their first choice would be a low partition wall. The prospective residents’ age group should be considered in designing the bedroom and the height of the partition wall; the correlation analysis revealed a positive relation (*p* = 0.137*, sig. = 0.046) between physical health of the residents and their preferred height of partition wall: healthier aged adults prefer a higher partition wall while less healthy ones prefer a lower partition wall. Moreover, the correlation also indicated that there is a significant negative correlation between residents’ age group and their preference as to the height of the partition wall: The desired height of partition walls decreases as their age increases. A possible explanation is that, in the event of an accident in the personal chamber, such as a fall or a heart attack, some aged adults would place a high value on an environment which permits the fastest possible response, so that the emergency could be seen by neighbors or by nurses. In this respect, notwithstanding the privacy issue, a lower partition wall has advantages over a high partition wall. Another study also supports this finding: Participants with hospitalization experience might be willing to accept a bed with reduced visual privacy, due to the concern of safety [30]. Moreover, the regression analysis indicated that both physical and mental health had a negative association with the height of the partition wall: the higher the partition wall, the lower the health score. The quadratic regression indicated that the optimal height for the partition wall was 1.85 m.

### 4.4. Unit Density (Number of People per Unit)

Crowding has a profound influence on privacy. The research revealed that occupants’ mental health self-report decreased when the number of residents per unit increased. Research consistently found that residents with two roommates experience greater anxiety than those with one [10]. Attempts have been made in nursing homes to create a more domestic feeling, and to foster a sense of personal belonging, by providing residents with individual units. Many countries advocate the provision of care on a smaller scale, characterized by more private rooms and baths [31] or small-house nursing homes [32] to eliminate their institutional atmosphere by creating a home-like setting.

Although the challenge for C&A homes in Hong Kong is exceptional in terms of the number of residents requiring accommodation and the very limited space available, independent personal bedrooms instead of hospital-like bedrooms should be provided to lower the density and improve the quality of life. When the condition is not allowed to provide individual bedrooms, there should be no more than seven people living in a multi-bed room.

### 4.5. Personal Control over Privacy

Research has shown that individuals feel safer and more comfortable when they can adjust their privacy level to suit the situation: for personal care, a chat with a neighbor or late-night reading [25]. This study revealed that personal control is a significant predicator for health status, because personal control may have stress-reducing effects. A movable barrier around the bed, or blinds over the window of the partition wall, can increase privacy when needed, and this is especially important for occupants in double or multi-bed rooms because the occupants shared the same space.

## 5. Conclusions

The creation of spaces with edges and control over privacy will help individuals to establish a sense of ownership [7]. However, the perception and need of privacy, on the other hand, might be culturally contextual. The provision of privacy for the elderly should be balanced with their needs for social interactions and open area. The bedroom should provide windows with a view of the outdoors, and a view of the indoors via an open area above the partition wall for communicating with neighbors or looking into the corridor for communicating with facility staff and family. The present study suggests that the desirable amount of open surface in each residential unit should be around 15 m^2^. Based on the collected data, the ideal openness/solid wall ratio in C&A homes is 0.57 (half of the surroundings are covered by partition wall). A balance must be achieved in terms of openness/solid wall ratio per bed, to maintain an adequate level of privacy and at the same time to allow occupants adequate social and psychological interactions.

There should be different types of bedrooms in aged healthcare facilities, with different heights of the partition wall. The preference of bedroom differs primarily according to age groups: younger residents may tend to choose higher partition wall chambers while senior residents may opt for lower partition wall chambers. The aged healthcare facilities should allow different heights of the partition wall to cater for personal preferences. In addition, based on the regression analysis of this study, the ideal height for the partition wall should be around 1.85 m.

Unit capacity should be carefully controlled to avoid overcrowding, which will influence the health and wellbeing of the residents. The linear and quadratic regression indicated that the number of people in each unit had a negative influence on the self-reported physical health status of the occupants in C&A homes. From the result of the quadratic regression analysis, there should be no more than seven people in each residential unit. Otherwise, the density of occupancy could lead to the decline of the physical health of the occupants.

The individual need to exercise control over the privacy of residents’ personal space should be taken into consideration in renovation or new design of bedrooms. In multi-bed rooms, each bed should be equipped with at least a movable curtain to protect the individual’s daily privacy.

The findings of this study could provide significant evidence to the Residential Aged Care Accreditation Scheme (RACAS) for C&A Homes in Hong Kong. In the future facility evaluation, privacy could be included as one of the criteria in the RACAS. Moreover, the findings of this study could benefit the disciplines of nursing care and the environmental psychology in the study of healthcare environments, which could promote the development of the aging industry in Hong Kong as well as worldwide. In addition, the findings of this study could be applied in crowed nursing homes in other Asian countries with high density such as Singapore.

## 6. Future Area of Research

This pilot study focusses on five parameters which could influence the privacy and health status of residents. Never-the-less, other parameters might also affect the privacy level such as acoustic isolation, visual penetration, and special arrangement in C&A homes. Future study could focus on these parameters. Moreover, due to limited time and resources, only nine C&A Homes were investigated in this pilot study. The types of chambers vary from one C&A Homes to another, and residents’ privacy preferences may differ according to the accommodation they have seen and experienced. In future studies, it would be advantageous to include more facilities and more respondents so that comprehensive guidelines may be developed. Gender difference shall be considered in future studies.

## Figures and Tables

**Table 1:** Descriptive analysis.

Items	Sample Size	Mean
Gender		
Male	74	35%
Female	139	65%
Age groups		
65–75 years	64	30%
75–85 years	59	28%
>85 years	90	42%
SF-12v2		
Physical Component Summary score (PCS)	213	41.8
Mental Component Summary score (MCS)	213	50.8

**Table 2:** Mean value of five parameters measured in C&A homes.

Parameter	R1	R2	R3	R4	R5	R6	R7	R8	R9
Total open surface per unit	11.51	15.57	10.44	8.88	8.79	15.28	14.41	6.79	8.79
Openness/solid ratio per bed	0.50	0.67	0.50	0.42	0.49	0.76	0.49	0.71	0.76
Height of partition wall	2.18	1.89	1.76	2.13	1.56	1.32	2.03	2.30	2.50
Unit density (number of people per unit)	1	1.6	1	1	1	7.8	1	5.7	4.3
Personal control over privacy	2	2	1.5	2	1.4	0.6	1.3	1.2	1.4

**Table 3:** Regression analysis between privacy and health status.

Parameter	Health Status	Equation	Sig.	Constant	b1	b2
Total open surface per unit	PCS	Linear	0.001	33.219	0.787	
		Quadratic	0.002	20.113	3.242	−0.107
Window/wall ratio per bed	PCS	Linear	0.010	50.562	−15.137	
		Quadratic	0.000	−81.352	447.095	−389.090
Height of partition wall	PCS	Linear	0.021	51.459	−4.803	
		Quadratic	0.000	−36.733	88.196	−23.805
	MCS	Linear	0.029	59.59	−4.390	
People/unit	PCS	Linear	0.000	45.040	−1.306	
		Quadratic	0.000	46.507	−2.722	0.190
Personal control	PCS	Linear	0.003	34.105	5.011	
		Quadratic	0.013	32.819	6.937	−0.664

## References

[1] Pedersen D.M. (1997). Psychological functions of privacy. J. Environ. Psychol..

[2] Westin A.F. (1968). Privacy and freedom. Wash. Lee Law Rev..

[3] Rapoport A. (1972). Some Perspectives on Human Use and Organization of Space.

[4] Altman I. (1975). The Environment and Social Behavior: Privacy, Personal Space, Territory, and Crowding.

[5] Lane P. (1990). A measure of clients’ perceptions about intrusions of territory and personal space by nurses. Meas. Nurs. Outcomes.

[6] Williams M.A. (1988). The physical environment and patient care. Ann. Rev. Nurs. Res..

[7] Hoglund J.D. (1985). Housing for the Elderly: Privacy and Independence in Environments for the Aging.

[8] Gou Z., Xie X., Lu Y., Khoshbakht M. (2018). Quality of life (qol) survey in hong kong: Understanding the importance of housing environment and needs of residents from different housing sectors. Int. J. Environ. Res. Public Health.

[9] Tate J.W. (1980). The need for personal space in institutions for the elderly. J. Gerontol. Nurs..

[10] Johnson F.L.P. (1979). Response to territorial intrusion by nursing home residents. Adv. Nurs. Sci..

[11] Kane R.A., Kling K.C., Bershadsky B., Kane R.L., Giles K., Degenholtz H.B., Liu J., Cutler L.J. (2003). Quality of life measures for nursing home residents. J. Gerontol. Ser. A Biol. Sci. Med. Sci..

[12] Applegate M., Morse J.M. (1994). Personal privacy and interactional patterns in a nursing home. J. Aging Stud..

[13] Leino-Kilpi H., Välimäki M., Dassen T., Gasull M., Lemonidou C., Scott A., Arndt M. (2001). Privacy: A review of the literature. Int. J. Nurs. Stud..

[14] Gou Z., Lau S.-Y.S., Lin P. (2017). Understanding domestic air-conditioning use behaviours: Disciplined body and frugal life. Habitat Int..

[15] Stokols D. (1972). A social-psychological model of human crowding phenomena. J. Am. Inst. Plan..

[16] Evans G.W. (2003). The built environment and mental health. J. Urban Health.

[17] Gabe J., Williams P. (1986). Is space bad for your health? The relationship between crowding in the home and emotional distress in women. Sociol. Health Illn..

[18] Lepore S.J., Evans G.W., Schneider M.L. (1991). Dynamic role of social support in the link between chronic stress and psychological distress. J. Personal. Soc. Psychol..

[19] Schopp A., Leino-Kilpi H., Välimäki M., Dassen T., Gasull M., Lemonidou C., Scott P.A., Arndt M., Kaljonen A. (2003). Perceptions of privacy in the care of elderly people in five european countries. Nurs. Ethics.

[20] De Chesnay M. (2014). Nursing Research Using Ethnography: Qualitative Designs and Methods in Nursing.

[21] Bäck E., Wikblad K. (1998). Privacy in hospital. J. Adv. Nurs..

[22] Tao Y., Gou Z., Lau S.S.-Y., Lu Y., Fu J. (2018). Legibility of floor plans and wayfinding satisfaction of residents in care and attention homes in hong kong. Australas. J. Ageing.

[23] Franz G. Space, Color, and Perceived Qualities of Indoor Environments. Proceedings of the 19th International Association for People-Environment Studies Conference (IAPS 2006).

[24] Indraprastha A., Shinozaki M. (2012). Computational models for measuring spatial quality of interior design in virtual environment. Build. Environ..

[25] Johnson C.A. (1974). Privacy as personal control. Man-Environment Interactions: Evaluations and Applications: Part 2.

[26] Barlas D., Sama A.E., Ward M.F., Lesser M.L. (2001). Comparison of the auditory and visual privacy of emergency department treatment areas with curtains versus those with solid walls. Ann. Emerg. Med..

[27] Lam E.T., Lam C.L., Fong D.Y., Huang W.W. (2013). Is the sf-12 version 2 health survey a valid and equivalent substitute for the sf-36 version 2 health survey for the chinese?. J. Eval. Clin. Pract..

[28] Ulrich R.S. (1984). View through a window may influence recovery from surgery. Science.

[29] Lu Y., Ossmann M.M., Leaf D.E., Factor P.H. (2014). Patient visibility and ICU mortality: A conceptual replication. HERD Health Environ. Res. Des. J..

[30] Lu Y., Cai H., Bosch S.J. (2017). Key spatial factors influencing the perceived privacy in nursing units: An exploration study with eight nursing units in Hong Kong. HERD Health Environ. Res. Des. J..

[31] Kane R.A., Lum T.Y., Cutler L.J., Degenholtz H.B., Yu T.C. (2007). Resident outcomes in small-house nursing homes: A longitudinal evaluation of the initial green house program. J. Am. Geriat. Soc..

[32] Cutler L.J., Kane R.A. (2009). Post-occupancy evaluation of a transformed nursing home: The first four green house^®^ settings. J. Hous. Elder..

